# Developmental Phase-Specific Molecular Signatures and Signaling Pathways in Cryptorchidism-Induced Testicular Damage

**DOI:** 10.3390/biom15111584

**Published:** 2025-11-11

**Authors:** Xinying Wang, Fuming Deng, Yijing Chen, Xiaonan Liu, Dian Li, Xiangliang Tang, Hongkun Lai, Qianlong Li, Wen Fu, Guochang Liu, Zhongzhong Chen, Tianxin Zhao

**Affiliations:** 1 Department of Urology, Guangzhou Women and Children’s Medical Center, Guangzhou Medical University, Guangdong Provincial Clinical Research Center for Child Health, Guangzhou 510623, China; xinxin170670@126.com (X.W.); hkkkkk0220@163.com (H.L.); lql02072023@163.com (Q.L.);; 2 Center for Reproductive Medicine, Guangzhou Women and Children’s Medical Center, Guangzhou Medical University, Guangzhou 510623, China

**Keywords:** cryptorchidism, surgically-induced model, testicular damage, phase-specific molecular processes, NF-κB signalling pathway, PI3K-Akt signalling pathway

## Abstract

Cryptorchidism, characterized by undescended testes, is associated with infertility and increased cancer risk through complex, multifactorial pathophysiological mechanisms involving interconnected alterations in testicular microenvironment, including but not limited to elevated temperature, hormonal dysregulation, altered vascular perfusion, and immune responses. These factors interact synergistically to drive testicular pathology. Using a surgically induced bilateral cryptorchid mouse model established at postnatal day 21 (PND21), we investigated phase-specific pathological mechanisms through analyses at prepubertal (PND35) and sexually mature (PND70) phases. Our transcriptome analysis revealed distinct molecular signatures at different developmental phases, with prepubertal cryptorchid testes showing 2570 differentially expressed genes predominantly enriched in immunoproteasome components and inflammatory pathways, while sexually mature testes exhibited 883 differentially expressed genes primarily related to extracellular matrix (ECM) remodeling and oncogenic pathways. Prepubertal molecular changes indicated immunoproteasome activation and inflammatory responses, whereas mature-phase alterations were characterized by ECM reorganization and fibrotic remodeling. Functional analysis demonstrated prepubertal enrichment in spermatogenesis regulation and interferon responses, while mature-phase signatures were associated with apoptosis, epithelial–mesenchymal transition, and inflammatory signaling cascades. Phase-specific oncogenic pathway correlations revealed distinct mechanisms: metabolic reprogramming and epigenetic regulation in prepubertal testes versus structural remodeling and invasion-related pathways in mature testes. Molecular validation confirmed elevated PI3K-Akt and NF-κB signaling at both developmental phases, identifying these as potential therapeutic targets. This first phase-resolved characterization of cryptorchidism pathology provides insights into developmental phase-specific mechanisms and suggests timing-dependent therapeutic strategies. Although differing from human congenital cryptorchidism in developmental timing and etiology, our surgically induced model recapitulates anatomical testicular malposition with multiple inseparable pathophysiological alterations, and the identified molecular signatures reflect integrated responses to the complex cryptorchid microenvironment.

## 1. Introduction

Cryptorchidism, characterized by undescended testes, affects 2–8% of male infants worldwide with significantly higher prevalence in preterm neonates, representing the most common congenital defect of the male reproductive system [1]. This condition presents both immediate developmental concerns and long-term reproductive sequelae, including increased risks of infertility and testicular germ cell tumors (TGCTs) [2]. The molecular basis for these outcomes has been investigated through both clinical and experimental approaches, revealing complex pathophysiological mechanisms extending beyond simple anatomical malpositioning.

Clinical studies have demonstrated that cryptorchidism significantly impairs spermatogenesis, with Hildorf et al. reporting compromised fertility potential in 20–25% of boys despite orchiopexy within the first year of life [3]. This observation suggests persistent molecular alterations transcending anatomical correction. Cryptorchidism involves complex and interrelated pathophysiological alterations resulting from ectopic testicular positioning in the abdominal cavity. The aberrant microenvironment encompasses multiple interconnected factors: elevated testicular temperature (approximately 2–4 °C higher than the scrotal environment, reaching 37 °C), altered vascular perfusion, modified immune milieu, and secondary hormonal dysregulation [4,5,6]. These factors do not act independently but interact synergistically to drive testicular pathology, with thermal stress representing one significant component of this multifactorial injury cascade. Robinson et al. have documented how this thermal differential disrupts specific temperature-sensitive processes during spermatogenesis [7], while Deng et al. demonstrated that heat stress impairs Sertoli cell function through metabolic reprogramming [8]. In humans, the consequences of the prolonged cryptorchid condition include germ cell apoptosis, quantitative reduction in spermatogonia, and qualitative defects in sperm production, evidencedevidenced by a 50–80% decrease in germ cell density in untreated cases and irreversible spermatogenic block observed in histopathological studies [9].

The pathophysiology of cryptorchidism involves multiple interconnected factors beyond thermal stress. Abdominal positioning of undescended testes results in altered vascular perfusion patterns, potentially leading to intermittent ischemia and compromised oxygen delivery [5,10]. Additionally, the altered anatomical position modifies the local immune microenvironment, with changes in immune cell populations and inflammatory mediator profiles that may contribute independently to testicular dysfunction [4]. These hemodynamic and immunological alterations interact synergistically with thermal stress to create a complex pathophysiological cascade.

At the molecular level, several key pathways have been implicated in cryptorchidism-induced testicular damage. Hu et al. demonstrated that heat stress activates the ubiquitin-proteasome system (UPS), inducing caspase-dependent apoptosis through P53 and Bcl-2 family member upregulation while concurrently disrupting miRNAs essential for germ cell viability [11]. These findings complement clinical observations by Wang et al., who reported elevated levels of ubiquitin carboxyl-terminal hydrolase 1 (UCHL1) in cryptorchid males, confirming UPS-mediated proteotoxicity as a significant contributor to testicular damage [12].

The relationship between cryptorchidism and testicular cancer involves mechanisms beyond simple thermal injury. The Testicular Dysgenesis Syndrome (TDS) hypothesis, as described by Li et al., proposes that cryptorchidism, impaired spermatogenesis, and testicular cancer may represent different manifestations of a common developmental disturbance during fetal life [13]. Hutson’s investigations provide compelling evidence that undescended testes experience disrupted apoptotic processes during gonocyte development, potentially allowing primordial germ cells to escape normal differentiation while accumulating genetic alterations [14]. This model is supported by Hanna and Einhorn’s observations that persistent gonocytes may transform into germ cell neoplasia in situ (GCNIS), the established precursor lesion for most testicular germ cell tumors [15]. However, Ornstrup et al. recently reported that men with testicular cancer exhibit increased morbidity across multiple health domains before cancer diagnosis, with patterns extending beyond what the TDS hypothesis alone explains, suggesting a multifactorial etiology requiring further investigation [16].

Current clinical management, as reviewed by Shiraishi et al., emphasizes surgical intervention (orchiopexy) before 18 months of age [17]. However, epidemiological data from Xu et al. indicate that approximately 10% of untreated cryptorchidism cases eventually develop testicular cancer despite surgical correction [18], highlighting the persistence of molecular alterations that transcend anatomical repositioning.

Animal models have significantly advanced our understanding of the molecular pathophysiology of cryptorchidism. Long et al. demonstrated hyperactivation of PI3K-Akt-mTOR signaling in cryptorchid rat testes and showed that suppressing this pathway through retinoic acid treatment improved autophagy and restored spermatogenesis [19]. Complementary work by Liu et al. identified dysregulation of NF-κB-mediated inflammatory signaling in cryptorchid mouse testes [20]. Ferragut Cardoso et al. characterized the temporal progression of testicular alterations following experimental cryptorchidism induction in rats, establishing a timeline for tissue damage and potential recovery [21]. Despite these advances, most experimental studies have focused on adult or unilateral cryptorchidism models, overlooking the prepubertal developmental period critical for germ cell maturation and establishment of the hypothalamic–pituitary–gonadal axis.

Significant interspecies differences exist in testicular physiology and pathological responses to cryptorchidism. Vikraman et al. characterized distinct patterns of testicular descent timing between rodents and humans [22], while Mahmud et al. demonstrated differences in gonocyte maturation patterns [23]. These physiological variations manifest in differing pathological outcomes. Spontaneousspontaneous germ cell tumors are exceedingly rare in rodents, with most lesions being degenerative rather than malignant, whereas humans with cryptorchidism have a well-documented increased risk of testicular germ cell neoplasia.

To address current knowledge gaps regarding developmental phase-specific molecular alterations in cryptorchidism, we established a bilateral cryptorchid mouse model through inguinal canal blockage at postnatal day 21 (PND21). This approach enabled investigation of cryptorchidism-induced testicular damage at two critical timepoints: the prepubertal phase (PND35) and sexually mature phase (PND70). By integrating transcriptomics, bioinformatics, and functional validation, we aimed to elucidate phase-specific molecular pathways characterizing the progression of cryptorchidism-induced testicular damage.

Our research identified distinct molecular signatures across developmental phases. Prepubertal prepubertal testes predominantly exhibited gene expression patterns associated with inflammatory responses and metabolic alterations, while sexually mature testes showed significant enrichment in pathways related to extracellular matrix organization and oncogenic signaling. Components of both PI3K-Akt and NF-κB signaling pathways showed elevated expression across developmental phases. While our surgically induced model creates anatomical malposition similar to cryptorchidism, it introduces multiple interconnected pathophysiological variables beyond temperature elevation alone. The observed molecular changes reflect the complex cryptorchid microenvironment rather than isolated thermal effects. Nevertheless, these findings enhance understanding of phase-specific molecular alterations in cryptorchid testes and may provide potential targets for therapeutic interventions tailored to different developmental phases, focused primarily on preserving fertility.

## 2. Materials and Methods

### 2.1. Animals and Experimental Cryptorchidism Model

Adult ICR mice were obtained from Beijing Huafukang Bioscience Co., Inc. (Beijing, China; Certificate No. SCXK 2019–0008, SPF grade). The animal experiments were sanctioned by the Institutional Animal Care and Use Committee (IACUC) at Guangzhou Huateng Biomedical Technology Co., Ltd.(Guangzhou, China). The mice were maintained under standardized environmental circumstances, with unrestricted access to normal laboratory chow and distilled water, a 12-h light/dark cycle, a regulated ambient temperature of 25 ± 2 °C, and a relative humidity of 50 ± 5%. After a one-week acclimation period, female mice were coupled with males at a 2:1 ratio, and the development of a vaginal plug on the same morning verified pregnancy, designated gestational day 0 (GD0). The health of the pregnant mice was systematically observed, and the exact birth dates of the progeny were documented.

Sixty male offspring with similar physiological circumstances at birth were randomly allocated to two experimental groups: the prepubertal phase group and the sexually mature phase group. Each experimental group was subdivided into a cryptorchidism subgroup (bilateral cryptorchidism surgically induced via inguinal canal occlusion) and a control subgroup (no surgical intervention). A bilateral cryptorchidism model was established by occluding the inguinal canal via an inguinal-scrotal surgical technique on postnatal day 21 (PND 21). The control group received no treatment. All the mice were individually housed in distinct cages on the basis of their designated experimental cohorts until their respective sampling time periods.

The prepubertal cohort was euthanized on postnatal day 35 (PND 35) for sample collection, with body weights recorded prior to euthanasia via cervical dislocation, an IACUC-approved technique. The left and right testes were excised, weighed, and recorded. The sexually mature group was subjected to a mating evaluation upon attaining sexual maturity, with samples taken on postnatal day 70 (PND 70) after the fertility examinations were completed. The harvested tissues were processed as follows: the left testis of each mouse was snap-frozen in liquid nitrogen for RNA and protein extraction, and the right testis was fixed in 4% paraformaldehyde (PFA) for histological examination. Additionally, portions of bilateral testes from all mice were collected and processed for ELISA analysis of proinflammatory (IL-1α/β, IL-6, IL-18, and TNF-α) and anti-inflammatory cytokines (G-CSF, IL-10, and TGF-β1/2/3) in both prepubertal (PND35) and sexually mature (PND70) phases, resulting in 30 testicular samples per group (bilateral testes from 15 mice per group). The epididymides were harvested for sperm enumeration and quality evaluation and were subsequently fixed in 4% PFA for histological analysis. All the experimental results, including body weight, testis weight, and sperm characteristics, are shown in Supplementary Table S1.

### 2.2. Hematoxylin-Eosin (HE) Staining

The testes from all the animals and the epididymides from the sexually mature mice were subjected to dehydration and paraffin embedding and subsequently sectioned into 4-μm slices. The tissue sections were subsequently deparaffinized, rehydrated, and stained with hematoxylin and eosin. Histological changes were analysed via a light microscope (ECLIPSECi-E, Nikon, Tokyo, Japan). The Image-Pro Plus 6.0 program (Media Cybernetics, Rockville, MD, USA) was utilized for quantitative analysis [24].

### 2.3. Reproductive Assessment

Sexually mature male mice, comprising both the control groups and cryptorchid mice, were cohabited with sexually mature female mice for a period of 10 days, with each male kept alongside two females. Subsequent to this cohabitation phase, the females were segregated and monitored for one month. The production of progeny by the female mice throughout this observation period confirmed successful mating, indicating the fertility of the corresponding male. A favourable mating result was documented under these circumstances.

### 2.4. Analysis of Sperm Quality

The methodology utilized for sperm quality analysis was modified from an existing protocol [25]. The cauda epididymis was excised from each mouse and precisely sectioned with microscissors. The excised tissue was then treated with 0.5 mL of a buffer solution containing 0.5% bovine serum albumin (BSA; Sigma, St. Louis, MO, USA) at 37 °C for 15 min to facilitate sperm release. Following incubation, the sperm samples were appropriately diluted and examined with the Hamilton Thorne Ceros II analytical equipment (Hamilton Thorne, Beverly, MA, USA). A minimum of six fields per sample were assessed to determine the sperm concentration and motility percentage.

### 2.5. Quantitative Real-Time Reverse Transcription Polymerase Chain Reaction (qRT-PCR)

Quantitative reverse transcription polymerase chain reaction (qRT-PCR) analysis was conducted on testicular tissue samples from nine randomly selected mice in each of the four experimental groups to evaluate the mRNA expression levels. Total RNA was isolated from the testes via TRI Reagent Solution (TR118, Ambion, Austin, TX, USA) following the manufacturer’s instructions. The RNA was subsequently reverse transcribed into complementary DNA (cDNA) via the RevertAid First Strand cDNA Synthesis Kit (K1622, Thermo Fisher Scientific, Waltham, MA, USA). Quantitative real-time PCR (qRT-PCR) was performed via the SYBR Select Master Mix Kit (catalogue no. 4472908; Thermo Fisher Scientific, Waltham, MA, USA) on an Applied Biosystems StepOnePlus Real-Time PCR System. The thermal cycling strategy included an initial denaturation phase at 95 °C for 10 min, followed by 40 amplification cycles of 10 s at 95 °C and 60 s at 60 °C. Gene expression levels were measured via the 2^−ΔΔCt^ method, with β-actin (ACTB) serving as the reference gene for normalization. The primer sequences are shown in Table S1. Each qRT-PCR experiment was conducted in triplicate, including both biological and technical repetitions.

### 2.6. Western Blotting

The Western blotting approach was performed following the methodology detailed in our prior paper [26]. The principal antibodies utilized were anti-PI3K (CST4249s, Cell Signaling Technology, Danvers, MA, USA), anti-phospho-Akt (CST4060S, Cell Signaling Technology, Danvers, MA, USA), anti-pan-Akt (CST4691 s, Cell Signaling Technology, Danvers, MA, USA), anti-P65 (GB11997-100, Servicebio), and anti-P50 (GB115431-100, Servicebio, Wuhan, China). Anti-β-actin (200068-6D7; Zen Bio, Chengdu, China) served as the loading control. Protein band visualization was conducted with the Super ECL Plus Kit (S6009, US Everbright Inc., Suzhou, China). The quantitative analysis of the bands was conducted via Image Lab software, version 5.2.1 (Bio-Rad, Hercules, CA, USA).

### 2.7. Quantification of Cytokines in Testicular Tissues

Cytokine concentrations (pg/mg) in testicular tissue samples, including granulocyte colony-stimulating factor (G-CSF), interleukin (IL)-1α, IL-1β, IL-6, IL-10, IL-18, tumor necrosis factor (TNF)-α, and transforming growth factor (TGF)-β1, β2, and β3, were measured via enzyme-linked immunosorbent assay (ELISA) kits (CSB-E04609m, CSB-E04621m, CSB-E08054m, CSB-E04639m, CSB-E04594m, CSB-E04609m, CSB-E04741m, CSB-E04726m, CSB-E09785m, and CSB-E12862m), all of which were sourced from CUSABIO, China. To make tissue homogenates, approximately 100 mg of testicular tissue was acquired from each sample, rinsed with phosphate-buffered saline (PBS), coarsely chopped, and subjected to ultrasonication. The supernatant was immediately collected for further examination. All procedures were executed in strict compliance with the manufacturer’s guidelines, as previously detailed in our paper [27]. In this investigation, bilateral testes from all mice in each experimental group (30 testicular samples per group, from 15 mice) were collected and processed, and each measurement was performed in triplicate.

### 2.8. Source of Data

Testicular transcriptome data were obtained from both prepubertal and sexually mature mice, with each developmental cohort comprising three cryptorchid and three control animals (*n* = 3 per group).

### 2.9. Differentially Expressed Genes (DEGs) and Enrichment Analysis

Differentially expressed genes (DEGs) were identified via the ‘limma’ package (version 3.44.3), applying the criteria of |logFC| > 1 and *p* value < 0.05, as detailed in reference [28]. Differentially expressed genes (DEGs) between cryptorchid and control groups were classified as Pre-DEGs for prepubertal mice and Mat-DEGs for sexually mature mice. A volcano plot was created via the R package ‘ggpubr’ (version 0.4.0; available at: https://github.com/kassambara/ggpubr, accessed on 22 November 2023). A heatmap was generated with the R package ‘pheatmap’ (version 1.0.12; available at: https://cran.r-project.org/package=pheatmap, accessed on 22 November 2023). Biological function and pathway analyses of differentially expressed genes (DEGs) were performed via Gene Ontology (GO) and Kyoto Encyclopedia of Genes and Genomes (KEGG) analyses, which were enabled by the R package ‘clusterProfiler’ (version 4.4.3) [29]. The R packages ‘clusterProfiler’ (version 4.4.3) and ‘org.Rn.eg.db’ (version 3.12.0) were utilized to conduct gene set enrichment analysis (GSEA) for predifferentially expressed genes (predEGGs) and postdifferentially expressed genes (Mat-DEGs). The hallmark gene sets and immunomic signature gene sets were obtained from the Molecular Signatures Database (MSigDB).

### 2.10. Identification of Key Genes

The protein-protein interaction (PPI) network for Pre-DEGs and Mat-DEGs was constructed via the Search Tool for the Retrieval of Interacting Genes (STRING) platform (https://string-db.org, accessed on 22 November 2023). The cytoHubba plug-in in Cytoscape (version 3.9.1) was subsequently utilized to identify hub genes within the PPI network through topological analysis [30]. This plugin was specifically employed to filter the complex PPI networks and identify the most critical central genes by ranking nodes according to their network topological properties. The key genes were identified via four independent topological algorithms implemented in cytoHubba: maximum cluster centrality (MCC), maximum neighborhood component (MNC), degree, and closeness. The top 20 genes identified by each method were intersected to ascertain the key genes, which included both Pre-key and Mat-key genes. This multi-algorithm approach enhances the robustness of hub gene identification by prioritizing genes that consistently rank highly across different network centrality measures. Ultimately, principal component analysis (PCA) was employed to assess the capacity of important genes to differentiate samples.

### 2.11. Gene Set Enrichment Analysis (GSEA) and Ingenuity Pathway Analysis (IPA)

GSEA enrichment analysis for Pre-key and Mat-key genes was performed via the R packages clusterProfiler (v4.4.3) and org.Rn.eg.db (v3.12.0). Hallmark and immunomic signature gene sets were obtained from the Molecular Signatures Database (MSigDB). We examined pathway intersections from GSEA data for prepubertal, sexually mature, and all marker genes individually. UpSet plots were created via the R package UpSetR to efficiently illustrate these crossings. The QIAGEN Ingenuity Pathway Analysis (IPA) tool (accessible at www.qiagen.com/ingenuity, accessed on 22 November 2023) was utilized to investigate disease and functional pathways in Pre-key and Mat-key genes, with a Z score > 2 signifying substantial activation and a Z score ≤ −2 signifying substantial inhibition.

### 2.12. Analysis of Correlations

Germ cell cancer (GC) genes were identified on the basis of a previously published study [31]. Specifically, genes listed in the Supplementary Table S1 and Data S3C (cluster 3) of reference [31] were utilized for this analysis. To assess the relationships between the identified key genes and GC genes, Spearman correlation analysis was employed to evaluate the associations between GC genes and Pre-key genes, with significantly correlated genes identified as Pre-GC genes. Gene Ontology (GO) and Kyoto Encyclopedia of Genes and Genomes (KEGG) enrichment analyses were subsequently performed on these Pre-GC genes. Similarly, Spearman correlation analysis was conducted to assess the relationships between GC genes and Mat-key genes, with significantly correlated genes designated Mat-GC genes. GO and KEGG enrichment analyses were then performed on these Mat-GC genes. Statistical significance for enrichment analyses was set at an FDR < 0.05.

### 2.13. Drug Prediction

The key genes were input into the Drug-Gene Interaction Database (DGIdb) to identify potential therapeutic agents for mitigating testicular injury in cryptorchidism. The interactions between the predicted drugs and key genes were then analyzed, and the outcomes were visualized using Cytoscape (version 3.9.1) [30].

### 2.14. Quantitative Analysis

The data were analysed with GraphPad Prism 9 software (GraphPad Software Inc., USA) and are presented as the mean ± standard deviation (SD). Statistical studies for quantitative outcomes, including body weight, testicular weight, testicular organ coefficient, pregnancy rate, semen analysis, qRT-PCR, Western blot (WB), and ELISA, were performed via unpaired *t* tests for parametric data and nonparametric testing when necessary. All bioinformatics studies were conducted via the R program. In the absence of alternative specifications, a *p* value of less than 0.05 was deemed statistically significant. Statistical significance for the experimental data was established as * *p* < 0.05, ** *p* < 0.01, and *** *p* < 0.001 compared with the control group. For the transcriptomic analysis, we acknowledge the limitation of a small sample size (*n* = 3 per group). With such sample sizes, the assumption of normality required for parametric tests like Student’s *t*-test cannot be reliably verified. Therefore, while we report *p*-values for differentially expressed genes using the ‘limma’ package as commonly practiced in exploratory transcriptomic studies, we emphasize the descriptive statistics (mean, standard deviation) and biological significance rather than relying solely on statistical significance. Fold-change magnitude and consistency of expression patterns were carefully considered in identifying key genes and pathways of interest.

## 3. Results

### 3.1. Morphological and Histopathological Alterations in Cryptorchid Testes at PND35 and PND70

**Figure 1:**
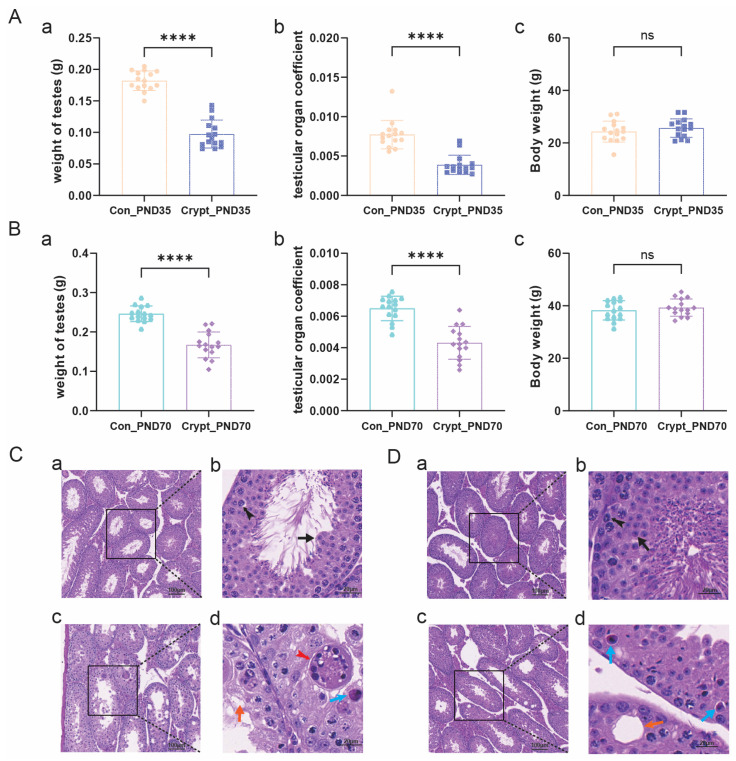
Impaired testicular development and histopathology in cryptorchidism. (**A**) Testicular morphometric analysis in prepubertal mice (PND35): (**a**) body weight; (**b**) testicular weight; (**c**) testicular index. (**B**) Testicular morphometric analysis in sexually mature mice (PND70): (**a**) body weight; (**b**) testicular weight; (**c**) testicular index. (**C**) Representative H&E staining of testicular sections from prepubertal mice. (**D**) Representative H&E staining of testicular sections from sexually mature mice. Arrows indicate primary spermatocytes (black), secondary spermatocytes (black split-tailed), multinucleated giant cells (red), vacuolated cells (orange), and degenerated cells (blue). Scale bars: 100 µm (**a**,**c**); 20 µm (**b**,**d**). *n* = 15 mice per group. Data are presented as mean ± SD. Statistical analysis: unpaired Student’s *t*-test. **** *p* < 0.0001 vs. control; ns, not significant. Abbreviations: Crypt, cryptorchidism; Con, control; PND, postnatal day.

The cryptorchid condition significantly impaired testicular development without affecting body weight in both prepubertal (PND35) and sexually mature (PND70) cryptorchid mice, as evidenced by a reduced testicular weight and testicular organ coefficient (Figure 1A,B). Histological analysis revealed phase-specific pathological changes: prepubertal cryptorchid testes presented disrupted seminiferous epithelium architecture with reduced epithelial layers, vacuolization, cellular degeneration, and multinucleated giant cell infiltration (Figure 1C). These structural alterations persisted into sexual maturity, characterized by sustained vacuolization, disorganized spermatogenic layers, and extensive cell degeneration (Figure 1D), whereas the control groups maintained normal testicular morphology throughout development. Comprehensive measurements of these parameters are presented in Supplementary Table S1.

### 3.2. Cryptorchidism-Induced Spermatogenic Dysfunction at PND70

**Figure 2:**
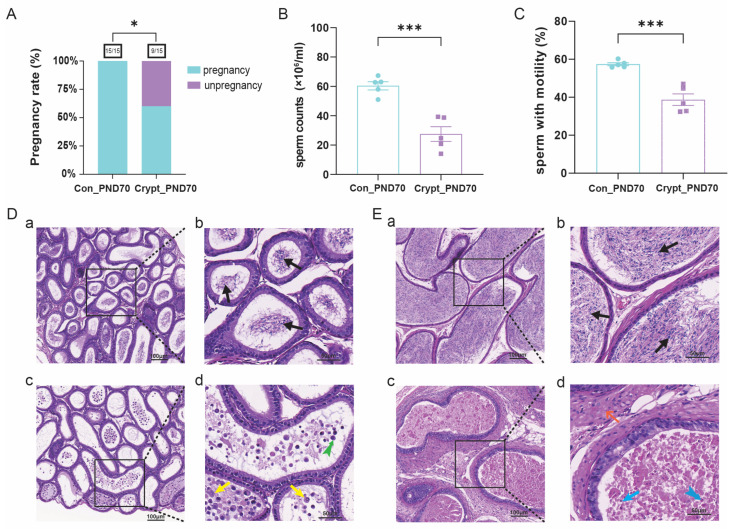
Cryptorchidism-induced spermatogenic dysfunction at PND70. (**A**) Fertility assessment in sexually mature mice (*n* = 15 per group). (**B**,**C**) Sperm parameters including sperm counts and sperm with motility (*n* = 5 per group). (**D**,**E**) Representative H&E staining of epididymal sections from prepubertal and sexually mature mice. The arrows indicate degenerated/necrotic cells (yellow), detached epithelial cells (green split-tailed), pyknotic nuclei (blue), karyorrhexis (blue split-tailed), and fibrotic interstitium (orange): Scale bars: 100 µm (**a**,**c**); 50 µm (**b**,**d**). Data are presented as mean ± SD. Statistical analysis was performed using unpaired *t*-test. * *p* < 0.05, *** *p* < 0.001 vs. control. Abbreviations: Crypt, cryptorchidism; Con, control; PND, postnatal day.

Assessment of reproductive function revealed significant impairment in cryptorchid mice, as evidenced by reduced pregnancy rates (Figure 2A), sperm counts (Figure 2B) and proportions of sperm with motility (Figure 2C). Histological analysis revealed phase-specific epididymal damage. Prepubertal cryptorchid mice presented inflammatory cell infiltration, epithelial cell exfoliation, and an absence of mature spermatozoa (Figure 2D), whereas sexually mature cryptorchid mice presented interstitial fibrosis, extensive cellular degeneration (karyopyknosis and karyorrhexis), and eosinophilic necrosis (Figure 2E). In contrast, the control groups maintained normal epididymal architecture with abundant luminal spermatozoa at both phases. Detailed quantitative data regarding reproductive parameters are provided in Supplementary Table S1.

### 3.3. Identification of Pre-DEGs and Mat-DEGs

Transcriptomic analysis revealed distinct gene expression patterns at different developmental phases in cryptorchid testes. In prepubertal testes, 2570 differentially expressed genes (Pre-DEGs) were identified (1050 upregulated, 1520 downregulated), whereas 883 DEGs (Mat-DEGs; 851 upregulated, 32 downregulated) were identified in sexually mature testes. Hierarchical clustering analysis revealed phase-specific transcriptional signatures, as visualized via heatmaps and volcano plots (Figure S1A–D). Comprehensive data for all identified differentially expressed genes are provided in Supplementary Tables S2 and S3.

### 3.4. Phase-Specific Pathway Enrichment Analysis

Functional enrichment analysis revealed both common and phase-specific pathway alterations. Both Pre-DEGs and Mat-DEGs were significantly enriched in temperature response pathways. Detailed information regarding these enrichment results can be found in Supplementary Tables S4–S7.

#### 3.4.1. Prepubertal Phase Characteristics

**Figure 3:**
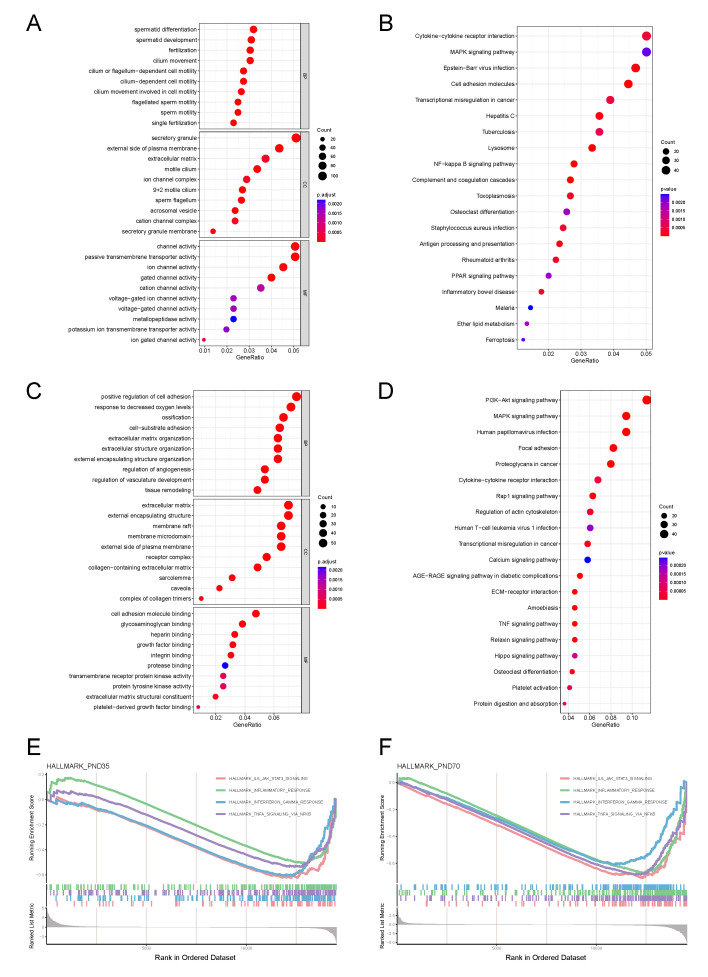
Phase-specific pathway enrichment analysis in cryptorchidism. (**A**,**B**) GO and KEGG pathway enrichment analysis of Pre-DEGs. (**C**,**D**) Corresponding analysis of Mat-DEGs. (**E**,**F**) Gene set enrichment analysis (GSEA) of Pre-DEGs and Mat-DEGs. *p* < 0.05 was considered significant.

The Pre-DEGs were predominantly enriched in spermatogenesis and inflammatory pathways (GO analysis; Figure 3A), including TLR signalling and cytokine production (IL-6/1β and IL-10). KEGG analysis revealed enrichment in cytokine-receptor interactions, cell adhesion pathways (Figure 3B), and key signalling cascades (NF-κB, PI3K-Akt, MAPK, PPAR, HIF-1, Ras, and JAK-STAT). Detailed information regarding these enrichment results can be found in Supplementary Tables S4 and S5.

#### 3.4.2. Sexually Mature Phase Characteristics

The Mat-DEGs presented distinct functional enrichment patterns. GO analysis revealed significant enrichment in reproductive processes, particularly gonadotropin responses and GnRH signalling. Cell cycle-related pathways, especially those related to G1/S transition and ERK1/2 cascade regulation, were notably enriched. Stress response pathways, including hypoxia-related processes and HIF-1 signalling, were significantly enriched. Inflammatory pathways, particularly the NF-κB and TNF signalling cascades, showed sustained activation. Additionally, oncogenic pathways, including the PI3K-Akt and Ras signalling pathways, were significantly enriched. Detailed information regarding these enrichment results can be found in Supplementary Tables S6 and S7.

GSEA revealed that inflammatory pathway enrichment (IL6-JAK-STAT3, IFN-γ, and NF-κB-TNF-α) was common between the two phases (Figure 3E,F). Detailed information regarding these enrichment results can be found in Supplementary Tables S8–S11.

### 3.5. Cryptorchidism-Activated Signalling Pathways and Inflammatory Mediators

**Figure 4:**
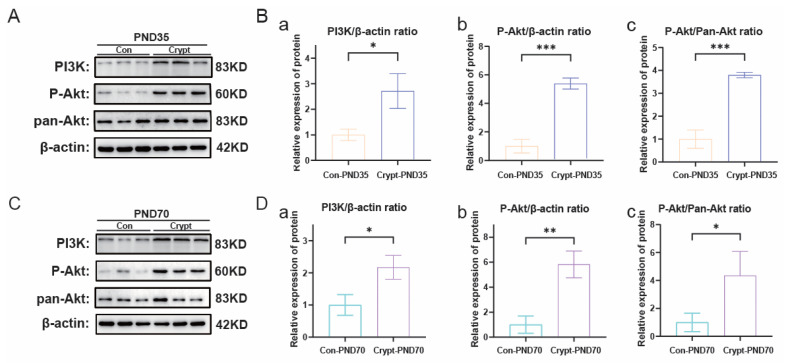
Cryptorchidism-induced activation of PI3K-Akt signaling. (**A**) Western blot analysis of PI3K, P-Akt, and Pan-Akt protein expression in prepubertal testes (PND35). (**B**) Quantification of PI3K-Akt pathway proteins in PND35 testes: (**a**) PI3K/β-actin ratio; (**b**) P-Akt/β-actin ratio; (**c**) P-Akt/Pan-Akt ratio. (**C**) Western blot analysis of PI3K, P-Akt, and Pan-Akt protein expression in sexually mature testes (PND70). (**D**) Quantification of PI3K-Akt pathway proteins in PND70 testes: (**a**) PI3K/β-actin ratio; (**b**) P-Akt/β-actin ratio; (**c**) P-Akt/Pan-Akt ratio. Data are presented as mean ± SD from three independent experiments. Statistical analysis: unpaired Student’s *t*-test. * *p* < 0.05, ** *p* < 0.01, *** *p* < 0.001 vs. the control. Abbreviations: Crypt, cryptorchidism; Con, control; PND, postnatal day.

**Figure 5:**
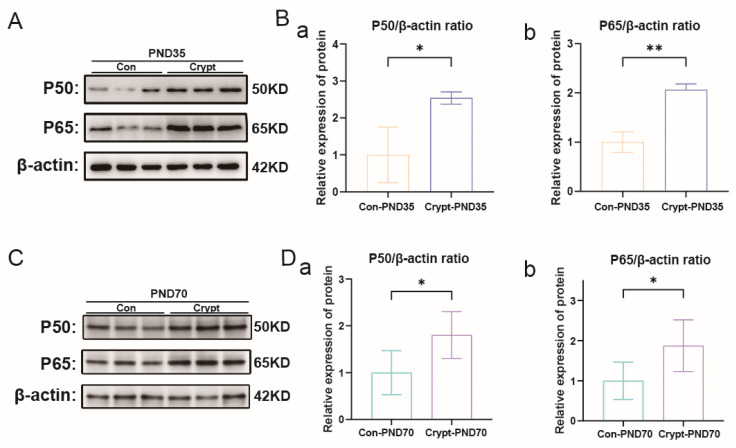
Cryptorchidism-induced activation of NF-κB signalling. (**A**) Western blot analysis of P50 and P65 protein expression in prepubertal testes (PND35). (**B**) Quantification of NF-κB subunits in PND35 testes: (**a**) P50/β-actin ratio; (**b**) P65/β-actin ratio. (**C**) Western blot analysis of P50 and P65 protein expression in sexually mature testes (PND70). (**D**) Quantification of NF-κB subunits in PND70 testes: (**a**) P50/β-actin ratio; (**b**) P65/β-actin ratio. Data are presented as mean ± SD from three independent experiments. Statistical analysis: unpaired Student’s *t*-test. * *p* < 0.05, ** *p* < 0.01 vs. the control. Abbreviations: Crypt, cryptorchidism; Con, control; PND, postnatal day.

Western blot analysis revealed sustained activation of PI3K-Akt signalling across developmental phases, as evidenced by elevated levels of PI3K, p-Akt, and pan-Akt in the cryptorchid groups (Figure 4A–D). Concurrent activation of NF-κB signalling was demonstrated by increased P50/P65 expression at both phases (Figure 5A–D). Comprehensive quantitative data of these signalling components are presented in Supplementary Material S1.

**Figure 6:**
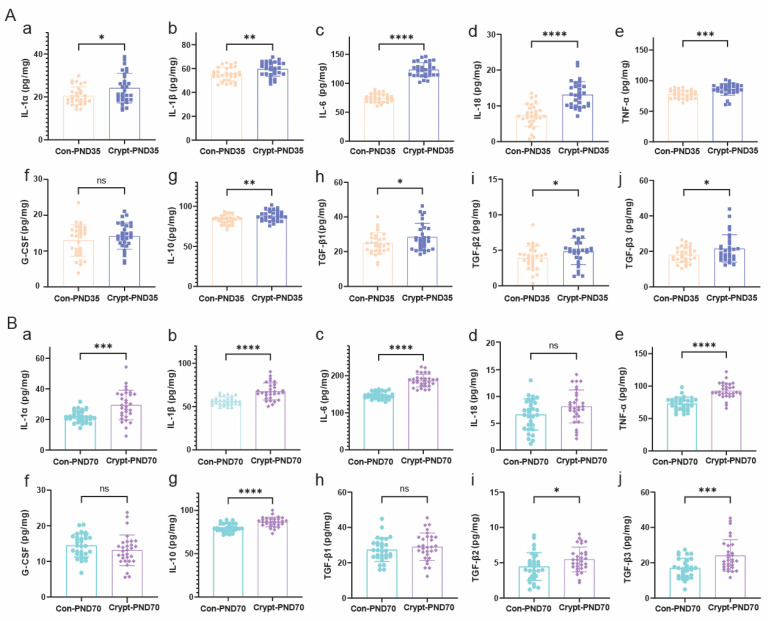
Phase-specific cytokine profiles in cryptorchid testes. ELISA quantification of cytokine levels in testicular tissues at (**A**) prepubertal (PND35) and (**B**) sexually mature (PND70) stages. Proinflammatory cytokines: (**a**) IL-1α; (**b**) IL-1β; (**c**) IL-6; (**d**) IL-18; (**e**) TNF-α. Anti-inflammatory cytokines: (**f**) G-CSF; (**g**) IL-10; (**h**) TGF-β1; (**i**) TGF-β2; (**j**) TGF-β3. Each group consisted of 30 testicular samples (bilateral testes from 15 mice per group). Data are presented as mean ± SD from three independent experiments. Statistical analysis: unpaired Student’s *t*-test. * *p* < 0.05, ** *p* < 0.01, *** *p* < 0.001, **** *p* < 0.0001; ns, not significant. Abbreviations: Con, control; Heat, heat-induced cryptorchidism; PND, postnatal day.

Phase-specific cytokine profiling revealed distinct inflammatory patterns. Prepubertal testes showed coordinated increases in both proinflammatory (IL-1β, IL-6, and TNF-α) and anti-inflammatory (IL-10) cytokines, with specific increases in IL-18 and TGF-β1 (Figure 6A). The sexually mature phase maintained elevated inflammatory mediator levels but exhibited normalized IL-18 and TGF-β1 levels (Figure 6B). G-CSF levels remained unchanged throughout development. Comprehensive quantitative data of these cytokine expression profiles are available in Supplementary Materials S2.

### 3.6. Identification and Validation of Phase-Specific Key Genes in Cryptorchidism

**Figure 7:**
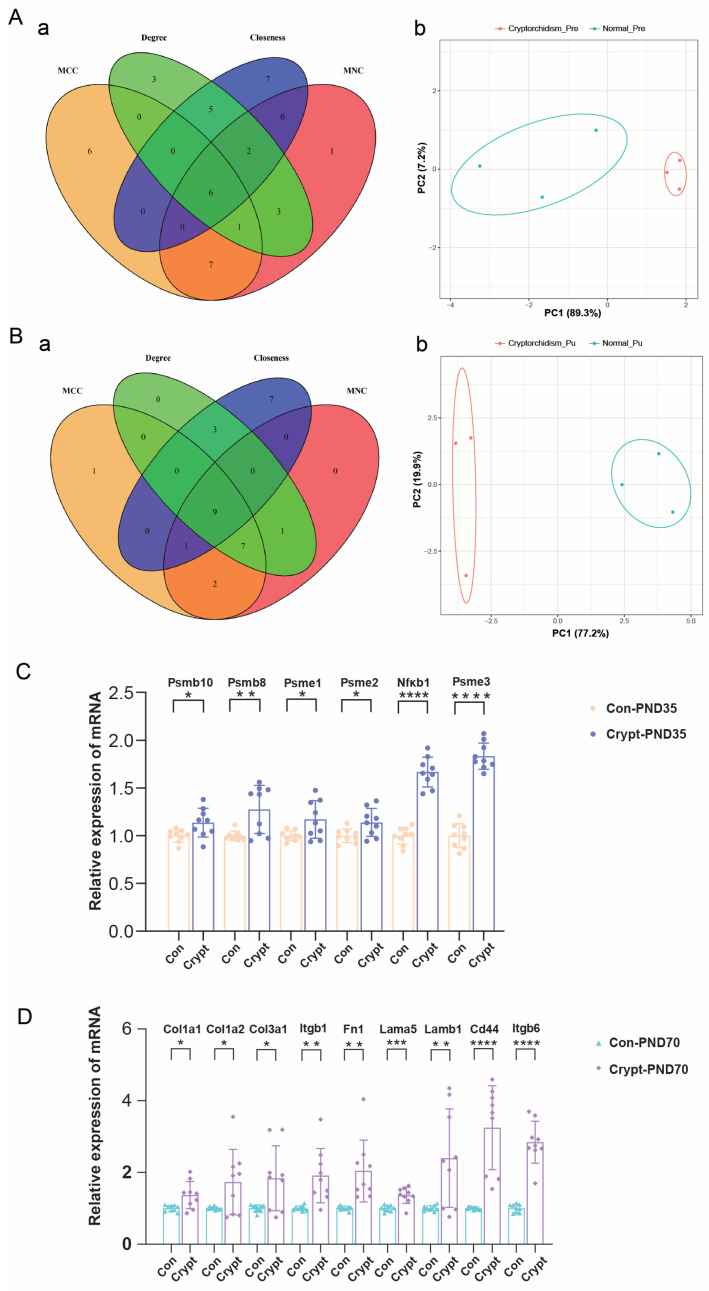
Identification and validation of phase-specific key genes in cryptorchidism. (**A**,**B**) Key genes identified by PPI network analysis and PCA clustering in prepubertal and sexually mature testes, respectively ((**a**) network analysis; (**b**) PCA plot). (**C**,**D**) qRT-PCR validation of key gene expression in prepubertal (PND35) and sexually mature (PND70) testes (*n* = 9 per group). The values represent the means ± SEMs from three independent experiments. Statistical analysis was performed using unpaired *t*-test. * *p* < 0.05, ** *p* < 0.01, *** *p* < 0.001, **** *p* < 0.0001 vs. the control. Abbreviations: Crypt, cryptorchidism; Con, control; PND, postnatal day.

Protein-protein interaction network analysis via multiple algorithms identified distinct phase-specific key genes. In prepubertal testes, six key genes were identified (*Psmb10*, *Psmb8*, *Psme1–3*, *Nfkb1*; Figure 7A(a)), which are involved primarily in proteostasis and inflammation, whereas in sexually mature testes, nine key genes (*Col1a1/2*, *Col3a1*, *Itgb1/6*, *Fn1*, *Lama5*, *Lamb1*, *Cd44*; Figure 7B(a)) associated with ECM remodelling were identified. Principal component analysis revealed a distinct separation between the cryptorchid and control groups at both phases (Figure 7A(b),B(b)). Comprehensive details of the Pre- genes and Mat-key genes identified through multiple algorithms (MCC, MNC, degree, and closeness) in the protein-protein interaction network analysis are presented in Supplementary Tables S12 and S13.

qRT-PCR validation confirmed significant upregulation of all identified key genes in the cryptorchid groups, with prepubertal testes showing elevated expression of immunoproteasome-related genes (Figure 7C) and sexually mature testes exhibiting increased levels of ECM-related genes (Figure 7D). Detailed quantitative expression data for these key genes at both developmental phases are available in Supplementary Material S3.

### 3.7. Functional Enrichment Analysis of Phase-Specific Key Genes

Pre-key genes (*Psmb10*, *Psmb8*, *Psme1*, *Psme2*, *Nfkb1*, and *Psme3*) were significantly enriched in spermatogenesis and interferon response pathways (Figure S2A). Conversely, the Mat-key genes were differentiated into two functional clusters: one group (*Col1a1*, *Col1a2*, *Col3a1*, *Lama5*, *Lamb1*, and *Itgb1*) was enriched primarily in apoptosis, interferon gamma response, and epithelial-mesenchymal transition (EMT) pathways, whereas the other cluster (*Fn1*, *Cd44*, and *Itgb6*) was associated predominantly with EMT, TNFA signalling via NF-κB, and mitotic spindle pathways (Figure S2B).

Notably, single-gene GSEA further identified 37 and 6 enriched pathways among the Pre-key and Mat-key genes, respectively (Figure S3A,B), with five shared inflammatory pathways, namely, the IL6-JAK-STAT3 signalling pathway, the inflammatory response pathway, and the TNFA-NFKB signalling pathway (Figure S3C), underscoring their significant associations with immune-related cellular processes and signalling cascades. Complete datasets from these enrichment analyses are provided in Supplementary Materials S4 and S5.

### 3.8. Association of Phase-Specific Key Genes with Oncogenic Pathways

**Figure 8:**
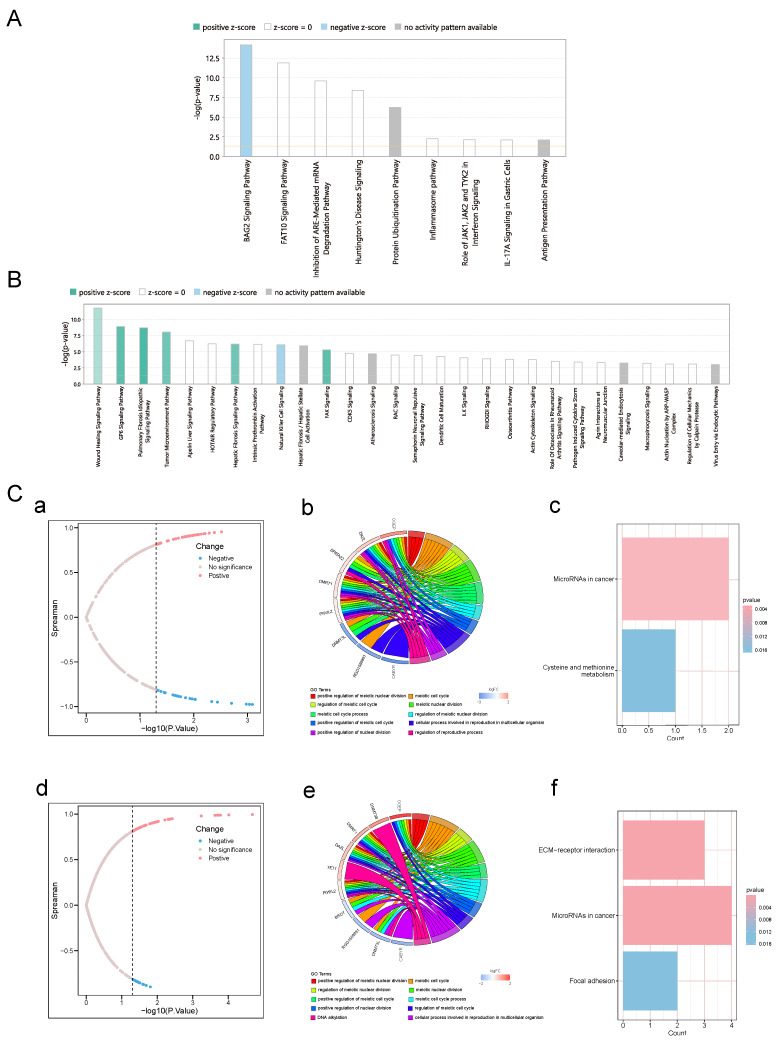
Phase-specific key genes and their associations with germ cell cancer pathways. (**A**,**B**) IPA-identified signalling pathways associated with Pre-key and Mat-key genes, respectively. (**C**) Correlation analysis with GC genes: identification and functional enrichment analysis of Pre-GC genes (**a**–**c**) and Mat-GC genes (**d**–**f**). (**a**,**d**) Gene correlation networks; (**b**,**e**) GO analysis; (**c**,**f**) KEGG pathway enrichment. Pathways with *p* < 0.05 were considered significant.

Ingenuity pathway analysis revealed distinct oncogenic pathway signatures between developmental phases (Figure 8A,B). In prepubertal testes, Pre-key genes showed selective pathway inhibition, most notably BAG2 signaling pathway suppression (Z-score = −2.1), alongside moderate activation of protein ubiquitination and EIF4 signaling pathways. In contrast, sexually mature testes exhibited broad oncogenic pathway activation, with Mat-key genes significantly upregulating multiple cancer-associated pathways including PI3K-Akt signaling, MAPK cascades, cell cycle regulation, and DNA repair mechanisms (Figure 8B). Correlation analysis with established germ cell cancer (GC) genes identified phase-specific oncogenic signatures with distinct mechanistic implications (Figure 8C). Prepubertal oncogenic associations centered on metabolic reprogramming and epigenetic regulation, with significant enrichment in cysteine/methionine metabolism and cancer-related microRNAs (Figure 8C(a–c)). Mature-phase oncogenic signatures focused on structural remodeling and invasion pathways, showing pronounced enrichment in ECM-receptor interactions, focal adhesion complexes, and microRNAs in cancer (Figure 8C(d–f)). These findings suggest that cryptorchidism-induced molecular alterations engage distinct oncogenic mechanisms depending on developmental timing.

The comprehensive correlation analysis data between key genes and germ cell carcinoma genes, including GO and KEGG enrichment analyses for both developmental phases, are extensively documented in Supplementary Materials S6.

### 3.9. Predicted Drug-Key Gene Interaction Analysis

Using the Drug-Gene Interaction Database (DGIdb), we identified potential drugs interacting with Pre-key and Mat-key genes. The drug–gene interaction network for Pre-key genes consists of 102 nodes and 108 edges (Figure S4A).

The drug network for Mat-key genes includes 27 nodes and 28 edges (Figure S4B).

Additionally, we used the Traditional Chinese Medicine Integrated Pharmacology Research Platform (TCMIP) to predict targeted herbal compounds for key genes (Figure S4C).

## 4. Discussion

In our surgically induced mouse model, bilateral cryptorchidism displaces testes to the abdominal cavity, potentially exposing testicular tissue to multiple microenvironmental alterations, including elevated temperature, vascular insufficiency, inflammatory activation, and metabolic perturbations. These interacting factors likely underlie the observed testicular atrophy, spermatogenic failure, and associated molecular alterations, reflecting the multifactorial cryptorchid microenvironment rather than thermal stress alone. In humans, cryptorchidism is associated with both impaired fertility and increased testicular cancer risk [1], though as discussed earlier, the incidence rates and age-related disease characteristics differ substantially between species. Due to ethical and practical constraints in obtaining testicular biopsies during orchiopexy procedures in children with cryptorchidism, which is not a routine clinical practice, most fundamental research on cryptorchidism has been conducted using experimental animal models. Recent molecular investigations have begun to elucidate heat-stress response mechanisms in testicular tissue. Zou et al. demonstrated that miR-128-3p modulates germ cell apoptosis under heat stress conditions [32]. Similarly, Chen et al. identified the upregulation of piR-020492 affecting AMPK and insulin signaling pathways during testicular hyperthermia [33]. While these studies have elucidated thermal stress mechanisms, the pathophysiology of cryptorchidism involves multifactorial alterations including thermal, vascular, immunological, and hormonal changes, yet the developmental phase-specific molecular signatures of cryptorchidism-induced testicular damage remain inadequately characterized. Our study addresses this critical knowledge gap through comprehensive phase-resolved analysis of cryptorchidism-induced testicular damage, revealing distinct molecular signatures across developmental phases. This approach has revealed that prepubertal testes predominantly exhibit immunoproteasome activation and inflammatory responses, while sexually mature testes show significant extracellular matrix remodeling and oncogenic pathway activation.

Our experimental design required careful consideration of both temporal and anatomical parameters to establish a clinically relevant yet methodologically robust cryptorchid model. We selected postnatal day 21 (PND21) for model induction based on multiple converging rationales: first, the technical constraints of neonatal mouse surgery, given their diminutive anatomical dimensions (average body weight <2 g) and heightened tissue vulnerability, preclude reliable surgical manipulation; second, PND21 represents a critical developmental milestone marking completion of the prepubertal period essential for normal hypothalamic–pituitary–gonadal (HPG) axis development [34]; and third, this timepoint provides an optimal experimental window to investigate cryptorchidism-induced testicular damage while addressing clinically relevant scenarios including consequences of delayed intervention and pathophysiology of acquired cryptorchidism (post-torsion or trauma). The selected timeframe enables systematic examination of progressive cryptorchidism-induced damage across two critical developmental phases—prepubertal (PND35) and sexually mature (PND70)—providing insights into phase-specific molecular signatures with direct therapeutic implications.

Regarding the bilateral versus unilateral model selection, we deliberately chose bilateral cryptorchidism despite its lower clinical frequency compared to unilateral presentations [35] for several methodological and scientific advantages. The bilateral design eliminates potential contralateral compensatory effects that could mask cryptorchidism-induced molecular alterations, thereby providing a more controlled system for therapeutic target identification. This approach is scientifically justified by clinical evidence from Hildorf et al., demonstrating that bilateral cryptorchidism cases exhibit significantly disrupted hormonal feedback mechanisms, with 23% of patients showing gonadotropin insufficiency characterized by low inhibin-B levels and reduced germ cell counts despite normal FSH levels [6]. This hormonal dysregulation indicates that bilateral involvement overwhelms normal compensatory mechanisms, potentially revealing molecular alterations masked in unilateral presentations where one descended testis maintains partial endocrine function. Clinical evidence further supports this selection: bilateral cryptorchidism cases demonstrate significantly reduced fertility rates compared to unilateral cases, where fertility approaches that of healthy males, with Cinislioglu et al. documenting higher incidence of epididymal anomalies in bilateral presentations [36]. These clinical observations directly correlate with our experimental findings of coordinated testicular and epididymal pathological alterations, indicating that our bilateral model accurately represents this more severe clinical phenotype and provides mechanistic insights into documented clinical outcomes. The core pathophysiological mechanism—prolonged supraphysiological temperature exposure, remains conserved regardless of laterality, ensuring that identified molecular pathways represent intrinsic cellular responses applicable to affected testes in both presentations. While Kowalska et al. reported no significant elevation in systemic inflammatory markers in human cryptorchid patients [37], this discrepancy with our findings of robust local inflammatory responses actually underscores the importance of tissue-specific analysis and supports the rationale for our bilateral model in detecting localized inflammatory processes that may be masked in systemic assessments. The bilateral model may thus provide a more stringent experimental platform for evaluating therapeutic interventions, following established preclinical development principles where treatments are tested under challenging conditions before clinical translation.

Our bilateral cryptorchid model demonstrated the gradual nature of reproductive tissue damage in the cryptorchid condition. The noted decrease in testicular mass and organ coefficients, with no significant changes in body weight, indicates a direct and specific effect of the cryptorchid condition on the development of reproductive tissues. Our histological analyses revealed distinct temporal patterns of tissue injury in cryptorchid testes. The prepubertal cryptorchid phase exhibited acute cellular responses, including epithelial disruption and inflammatory cell infiltration, whereas the sexually mature cryptorchid phase demonstrated enduring architectural disorganization despite diverse cellular pathologies. Significantly, whereas earlier research predominantly concentrated on cryptorchid testicular pathology [38], our phase-resolved study encompassed the cryptorchid epididymis, revealing coordinated destruction across the male reproductive system. The cryptorchid epididymal pathology clearly exhibited phase-specific development, ranging from initial epithelial disruption to subsequent fibrotic remodelling. The synchronized pathological alterations in cryptorchid epididymal tissues offer novel insights into the entire impact of cryptorchidism on male reproductive function. This systematic investigation delineates a temporal framework for cryptorchidism-induced reproductive tissue damage, emphasizing the necessity of incorporating both testicular and epididymal pathology in comprehending the progression of cryptorchidism-related reproductive dysfunction.

To further understand the molecular basis underlying these observed histopathological changes, we developed a phase-resolved approach to studying cryptorchidism that builds upon and extends previous methodological advances in the field. Hadziselimovic et al. established foundational gene expression patterns in cryptorchid tissue through genome-wide analysis [39], while Zhou et al. employed bioinformatics to identify critical regulatory pathways in cryptorchidism-affected spermatogenesis [40]. Most experimental studies on adult cryptorchidism have relied on controlled heat exposure models [41]. While these models are informative, they typically capture only static responses at specific time points rather than the dynamic progression of cryptorchidism-related pathological changes. More recently, single-cell transcriptome investigations have illuminated the critical role of testicular somatic cells in cryptorchidism progression, notably highlighting the contribution of mast cells in mediating fibrotic remodeling via TGF-β1 signaling in cryptorchid testes [4]. Our developmental phase-resolved approach enhances these findings by documenting the dynamic progression from prepubertal inflammation to mature-phase fibrosis in cryptorchidism. This temporal perspective reveals that cryptorchid testicular injury involves not only germ cell loss but also progressive microenvironmental remodeling, suggesting a more complex pathogenic process than previously recognized. The identification of phase-specific molecular signatures in cryptorchidism provides a framework for understanding how acute inflammatory responses evolve into chronic tissue remodeling, potentially guiding more tailored therapeutic strategies based on developmental timing of intervention for cryptorchid patients.

Applying the phase-resolved approach described above, our detailed transcriptomic analysis quantified the molecular differences between developmental phases. The transcriptomic profiles revealed striking phase-specific molecular signatures in cryptorchid tissue. We identified 2570 DEGs in prepubertal cryptorchid testes versus 883 DEGs in sexually mature cryptorchid testes, suggesting a more dramatic molecular dysregulation during early development in response to cryptorchidism. This finding carries significant clinical implications, reinforcing the importance of early intervention in cryptorchidism, consistent with clinical studies showing improved outcomes following early surgical correction [17]. The prepubertal period may represent a critical window for therapeutic intervention before permanent fertility damage occurs in cryptorchid patients, challenging the current paradigm that focuses primarily on anatomical correction without addressing underlying molecular alterations induced by cryptorchidism.

Our differential gene expression analysis and protein–protein interaction network analysis identified significant upregulation of key genes related to immunoproteasome components (*Psmb8*, *Psmb10*, *Psme1-3*) and *Nfkb1* in prepubertal cryptorchid testes, while pathway enrichment analysis, Western blot analysis, and ELISA analysis collectively confirmed the activation of NF-κB signaling cascade and elevated expression of inflammatory cytokines in the cryptorchid condition. These findings demonstrate a coordinated immune-inflammatory response in early-phase cryptorchid testes. While thermal stress from elevated temperature may contribute to these responses, the observed immunoproteasome activation and inflammatory signaling likely represent integrated cellular adaptations to the multifactorial altered microenvironment in cryptorchidism, potentially including hormonal dysregulation and hypoxic stress.

The immunoproteasome system has been extensively documented as an adaptive mechanism maintaining protein homeostasis and regulating inflammatory signals [42,43]. This specialized form of the proteasome is typically induced under stress conditions and plays crucial roles beyond protein degradation [44,45]. Specifically, previous studies have demonstrated that dysregulation of immunoproteasome components identical to those we identified (*Psme1-3* and *Psmb8/10*) directly contributes to inflammatory processes through enhanced antigen processing and modulation of pro-inflammatory cytokine production [46,47].

Our comprehensive analysis revealed sustained activation of both PI3K-Akt and NF-κB signaling pathways across developmental phases in cryptorchid testes, establishing them as fundamental mediators of cellular responses to the cryptorchid condition and promising therapeutic targets with distinct intervention windows. The PI3K-Akt pathway demonstrated multifaceted functions extending beyond inflammation regulation to broader cellular stress responses, as evidenced by its regulation of NF-κB activity and inflammatory cytokine production in prepubertal cryptorchid testes [48], and its influence on autophagy and apoptosis through PI3K/AKT/mTOR signaling under hypoxic conditions [49]. These findings align with and extend recent work by Jia et al., who identified PI3K-Akt signaling as critically enriched in cryptorchidism with AKT3 serving as a potential biomarker for azoospermia [50], and Long et al., who demonstrated that hyperactivated PI3K-Akt-mTOR signaling suppression via retinoic acid improves autophagy and restores spermatogenesis [19]. The clinical relevance of this pathway is supported by Cannarella et al.’s demonstration that IGF system interconnects with PI3K/AKT signaling in mediating FSH effects on Sertoli cells [51], suggesting that dysregulation of these pathways may represent a potential mechanism underlying the progressive reproductive impairment in cryptorchidism. This relationship is additionally supported by Centola et al.’s finding that FSH-induced Sertoli cell proliferation requires PI3K/Akt/mTORC1 pathway activation [52].

The precise molecular linkages among these pathways necessitate additional research, but recent studies indicate that PI3K-Akt signalling may influence inflammatory responses via NF-κB-dependent processes [53]. This cross-talk between signaling pathways is particularly relevant in cryptorchidism-induced testicular dysfunction, as demonstrated by Yadav et al., who reported that chronic unpredictable stress disrupts testicular function by perturbing the Nrf2/HO-1/IKKβ/NF-κB oxido-inflammatory pathway [54]. Collectively, these findings suggest that immunoproteasome activation and inflammatory responses may establish a self-perpetuating ‘proteotoxic stress-inflammation cycle’ in prepubertal cryptorchid testes, though this potential mechanism warrants further investigation.

The transition to extracellular matrix (ECM) remodeling in sexually mature cryptorchid testes represents a pivotal finding with significant implications for understanding disease progression and therapeutic intervention. Our comprehensive transcriptomic profiling identified a distinct mature-phase molecular signature dominated by ECM-related genes (*Col1a1*, *Col1a2*, *Col3a1*, *Itgb1/6*, *Cd44*, *Fn1*, *Lama5*, *Lamb1*), validated by qRT-PCR, which fundamentally differs from the prepubertal inflammatory phenotype. This developmental shift toward ECM dysregulation encompasses multiple interconnected processes: GO enrichment analysis revealed significant enrichment in endocrine-related pathways (particularly GnRH signaling) complementing clinical observations of hormonal imbalances [55], while pathway analyses demonstrated enrichment in cell–substrate adhesion, ECM organization, and tissue remodeling, with KEGG analysis highlighting HIF-1 pathway and ECM-receptor interaction categories.

Critically, it is essential to distinguish ECM remodeling as an adaptive tissue repair response rather than a direct oncogenic driver in our experimental model. While ECM components can contribute to tumor microenvironment remodeling in cancer contexts [56], their upregulation in cryptorchid testes likely reflects the complex pathological alterations associated with cryptorchidism, such as thermal stress from elevated temperature, potential alterations in blood supply, and hypoxic microenvironment [57]. These ECM changes appear to represent adaptive responses to tissue damage rather than direct drivers of malignancy, particularly given that testicular germ cell tumors typically involve germ cell-specific alterations rather than stromal ECM changes [58,59]. These findings gain mechanistic significance when interpreted alongside studies demonstrating that hypoxic conditions in cryptorchid testes enhance ECM formation through TGF-β/Smad signaling [57], suggesting a self-perpetuating cycle where the cryptorchid condition triggers progressive structural remodeling. The molecular transition from prepubertal inflammation to mature-phase ECM reorganization identifies specific components as potential therapeutic targets, with distinct intervention windows where early anti-inflammatory approaches may prevent progression to irreversible fibrotic changes that extend beyond the initial thermal insult.

The phase-specific molecular signatures we identified provide insights into potential mechanisms underlying cryptorchidism-induced testicular damage. Our transcriptomic analysis revealed distinct developmental phase-dependent alterations. Prepubertal testes showed significant activation of metabolic reprogramming pathways (particularly cysteine/methionine metabolism) potentially promoting oxidative stress and genomic instability [60,61], while sexually mature testes exhibited predominant upregulation of ECM remodeling genes. Although ECM components (including specific collagens such as *Col1a1/2*, *Col3a1*) have shown upregulated expression in some human malignancies and tumor microenvironments [56,62], in our rodent model these ECM changes likely represent adaptive responses to thermal stress rather than pre-neoplastic events. This interpretation requires careful consideration of the experimental context.

As established in the Introduction, cryptorchidism-associated cancer risk involves multifactorial mechanisms. In interpreting our findings of activated oncogenic pathways (PI3K-Akt, NF-κB) and phase-specific cancer-associated gene signatures, these molecular alterations should be understood as integrated cellular responses to the complex cryptorchid microenvironment rather than evidence of thermal-stress-induced oncogenesis.

In the context of human testicular cancer development, the sustained activation of these pathways warrants particular attention. In humans, cryptorchidism-associated testicular germ cell tumors (TGCTs) develop through a well-characterized pathological progression, beginning with the persistence of undifferentiated gonocytes beyond their normal developmental window. These persistent gonocytes are critical to TGCT pathogenesis, as they retain embryonic stem cell-like properties, express pluripotency markers, and fail to undergo normal differentiation into spermatogonia during early postnatal development [63]. In the normal testis, gonocytes migrate to the basement membrane and differentiate into spermatogonial stem cells shortly after birth; however, in cryptorchid conditions, the supraphysiological temperature and altered microenvironment may interfere with this transition, allowing gonocytes to persist inappropriately [64]. These persistent gonocytes may subsequently transform into germ cell neoplasia in situ (GCNIS), which serves as the precursor lesion for invasive tumors, particularly seminomas [65]. Recent molecular studies by Kilic et al. have further elucidated this pathogenesis, showing that abnormal gonocytes settle in the ‘spermatogonial niche’ under the influence of developmental factors and parental genomic imprinting erasure. These cells develop into pre-GCNIS cells through TPSY and OCT4 gene regulation, followed by whole genome duplication events leading to GCNIS formation [66]. Notably, both PI3K-Akt and NF-κB signaling have been implicated in this malignant transformation process, potentially through maintaining pluripotency factor expression (including OCT3/4, NANOG) and promoting anti-apoptotic mechanisms in persistent gonocytes [67,68,69]. Recent evidence suggests that constitutive activation of PI3K-Akt signaling may facilitate GCNIS development by preventing normal gonocyte differentiation while simultaneously supporting cell survival under thermal stress [70]. Similarly, aberrant NF-κB activation could contribute to the inflammatory microenvironment that supports GCNIS persistence and progression to invasive seminoma [71].

The phase-specific oncogenic pathway signatures we identified provide mechanistic insights into how cryptorchidism-associated molecular alterations may contribute to cancer risk through distinct temporal mechanisms. The prepubertal signature, characterized by metabolic reprogramming (particularly cysteine/methionine metabolism) and cancer-related microRNA dysregulation, aligns with established models of early oncogenic priming where metabolic alterations create cellular environments conducive to transformation [72,73]. Cysteine/methionine metabolism dysregulation is particularly concerning as these pathways regulate DNA methylation, oxidative stress responses, and cellular redox homeostasis [74], all of which areall critical determinants of genomic stability during the vulnerable prepubertal period when gonocytes undergo critical differentiation decisions [75].

These ECM-mediated structural changes, as previously detailed, represent a fundamental shift in the pathological landscape of cryptorchid testes during sexual maturation. The temporal evolution of molecular signatures, from metabolic dysregulation and immune activation to structural remodeling, provides insights into the progressive nature of cryptorchidism-induced testicular damage and highlights the importance of phase-specific therapeutic approaches.

The phase-specific inflammatory profiles, characterized by coordinated cytokine elevations in prepubertal testes and persistent inflammatory mediators in mature testes, align with mechanisms by which chronic inflammation may contribute to tissue remodeling and potentially tumorigenesis in prolonged human pathology [76]. Multiple factors including genetic predisposition, hormonal influences, and environmental exposures likely contribute to the species-specific and individual variations in response to cryptorchidism. When interpreting the oncogenic pathway associations in our results, it is crucial to recognize that these signaling cascades exhibit context-dependent functions and are frequently activated during tissue repair processes. Their presence in our model most likely reflects adaptive stress responses to the multifactorial cryptorchid microenvironment rather than direct neoplastic transformation, as the biological outcome is determined by the specific cellular context and temporal activation patterns. Therefore, while our findings identify molecular pathways that may contribute to long-term cancer risk in the context of persistent cryptorchidism in humans, they should not be interpreted as evidence that thermal stress alone drives malignant transformation. Future studies incorporating these variables would enhance understanding of how these molecular signatures relate to pathological outcomes in clinical contexts.

Based on these pathway analyses, phase-specific therapeutic interventions targeting these cascades may optimize treatment outcomes. The prepubertal inflammatory phase presents opportunities for early intervention using established anti-inflammatory approaches, while the mature-phase tissue repair mechanisms require targeted anti-fibrotic strategies. For prepubertal cryptorchidism, proteasome inhibitors represent a potential therapeutic avenue, with bortezomib and oprozomib demonstrating anti-inflammatory properties in other disease contexts [77,78] that suggest potential for reducing testicular damage through suppression of inflammatory gene expression networks. However, their application would require careful dose optimization and safety evaluation in pediatric populations. PI3K-Akt pathway modulation offers potential through mTOR inhibitors such as sirolimus (rapamycin), which has demonstrated anti-fibrotic properties in other organ systems [79]. The mature-phase ECM remodeling signatures suggest utility for anti-fibrotic interventions, though the limited number of targeted therapies (27 potential compounds versus 102 for prepubertal phase) reflects the specialized nature of these pathways. Traditional Chinese medicine approaches identified in our analysis may provide additional options for personalized treatment strategies.

Several important limitations must be acknowledged when interpreting our findings in the context of human cryptorchidism pathology. First, fundamental interspecies differences exist between rodent and human testicular physiology and pathological responses to cryptorchidism. Spontaneous germ cell tumors are exceedingly rare in rodents, with our model primarily exhibiting degenerative rather than neoplastic changes, a crucial distinction when considering human cryptorchidism-associated cancer risk. Rodents exhibit fundamentally different cellular responses to supraphysiological temperatures, with gonocytes typically undergoing apoptosis rather than the neoplastic transformation observed in human cryptorchidism [80]. Despite detecting activated PI3K-Akt and NF-κB pathways in our model, these pathways likely serve primarily stress-adaptive functions in rodents rather than the oncogenic roles they may play in human pathophysiology.

Beyond interspecies differences, a fundamental limitation inherent to our surgically induced cryptorchidism model is the inability to isolate individual pathophysiological factors contributing to testicular damage. While we hypothesize that our findings likely represent pathological and molecular alterations associated with thermal stress in cryptorchidism, and our model was specifically designed to recapitulate the intra-abdominal testicular positioning characteristic of human cryptorchidism, this experimental approach inevitably introduces multiple interconnected physiological perturbations beyond temperature elevation alone. The surgical creation of bilateral cryptorchidism through inguinal canal occlusion simultaneously introduces multiple interconnected physiological perturbations: elevated testicular temperature secondary to abdominal positioning, disrupted hypothalamic–pituitary–gonadal axis regulation affecting FSH, LH, and intratesticular testosterone homeostasis, compromised vascular perfusion with potential ischemic stress, altered immune microenvironment resulting from anatomical repositioning, and modified oxygen tension with consequent metabolic adaptation [57,81]. These variables are inherently confounded and cannot be experimentally dissociated in our model, precluding definitive attribution of the observed transcriptomic alterations, inflammatory responses, ECM remodeling, or signaling pathway activations to any single factor, including supraphysiological temperature exposure alone. The molecular signatures we identified therefore represent integrated cellular responses to the multifactorial cryptorchid condition rather than isolated effects of individual components.

The absence of persistent gonocytes and germ cell neoplasia in situ (GCNIS) in our experimental model represents a significant limitation, as these are critical precursor events in human testicular germ cell tumor development. While our transcriptomic analysis revealed altered metabolic and ECM remodeling patterns that parallel some changes observed in human testicular cancer microenvironments [82], the molecular alterations we observed may represent only early events in a complex pathological process. Clinical evidence indicates that malignant transformation in human cryptorchidism typically develops over extended timeframes, with approximately 10% of untreated cases eventually developing testicular cancer [18], a timeline and outcome not replicated in rodent models.

Additionally, our postnatal induction model at PND21 cannot fully recapitulate the effects of disrupted embryonic testicular descent that characterizes congenital human cryptorchidism. The differences in developmental timing, spermatogenic organization, and thermal stress responses between species limit direct translational interpretation. While our surgically induced model provides valuable insights into molecular alterations associated with cryptorchidism, it represents an acquired condition rather than the complete congenital developmental anomaly observed in human patients. These species-specific differences underscore that our findings should be considered as foundational insights into cryptorchidism-associated responses in mammalian testicular tissue rather than direct predictions of human cryptorchidism outcomes, warranting validation in human specimens and clinical studies for translational application.

A methodological limitation in our study is the relatively modest sample size (*n* = 3 per group) used for transcriptomic analysis, which, although common in exploratory transcriptomic investigations, warrants statistical consideration. With such limited replication, statistical assumptions such as normality distribution cannot be reliably tested, potentially affecting the robustness of *p*-value-based inferences. We have addressed this limitation by emphasizing effect sizes (fold-changes), biological coherence of gene expression patterns, and validation of key findings through complementary approaches (qRT-PCR, Western blotting, and ELISA). Future studies with larger sample sizes would enhance the statistical power and further validate the molecular signatures identified in our exploratory analysis.

Future translational research should focus on: (1) timing-dependent interventions that align with developmental phase-specific molecular signatures, (2) combination approaches targeting both survival and inflammatory pathways, and (3) biomarker development using the phase-specific gene signatures we identified. The distinct molecular profiles suggest that age-specific and molecularly guided therapeutic approaches may optimize treatment outcomes, though validation in human cryptorchidism models remains essential for clinical translation.

The translation of our molecular findings to clinical practice requires validation in human cryptorchid specimens with comprehensive assessment of testicular function beyond fertility outcomes, including testosterone production, inflammatory responses, and tissue atrophy. A key limitation of our tissue analysis is the inability to attribute differential expression patterns to specific testicular cell types. Future investigations should address this constraint through comprehensive single-cell approaches designed to answer fundamental questions in testicular biology. Single-cell RNA sequencing (scRNA-seq) will specifically address several critical knowledge gaps: (1) cell-type specific temperature sensitivity profiling to definitively identify which testicular cell populations exhibit the greatest vulnerability to supraphysiological temperatures, (2) mechanistic characterization of temperature-sensitive spermatogenesis by identifying specific molecular checkpoints where elevated temperature disrupts germ cell development, (3) temporal resolution of developmental phase-specific differences in spermatogenic programs and their differential temperature sensitivities, (4) dynamic immune cell recruitment characterization to map spatiotemporal patterns of inflammatory responses, and (5) spatial transcriptomics integration to maintain tissue architecture context while achieving single-cell resolution. These approaches will distinguish between compositional changes and intrinsic gene expression alterations within specific cell populations, addressing the fundamental interpretive challenge in bulk tissue analysis while enabling comparative analysis of unilateral and bilateral pathophysiological differences. Nevertheless, the consistency between our transcriptomic data and protein-level validations supports the biological relevance of our findings despite these statistical and methodological limitations.

## 5. Conclusions

In summary, our phase-resolved analysis revealed distinct molecular signatures of cryptorchidism-induced testicular damage across developmental phases. Prepubertal cryptorchid testes predominantly exhibited immunoproteasome activation, inflammatory responses, and metabolic reprogramming, while sexually mature testes showed prominent ECM remodeling gene upregulation, particularly specific collagens. Phase-specific inflammatory profiles were evident, with coordinated pro- and anti-inflammatory cytokine elevations in prepubertal testes, contrasting with persistent inflammatory mediator elevation in mature testes. The consistent activation of PI3K-Akt and NF-κB signaling pathways across developmental phases suggests they likely play important roles in cryptorchidism pathophysiology. While our surgically induced model at PND21 differs fundamentally from human congenital cryptorchidism in origin, timing, and pathological outcomes, it provides valuable insights into molecular alterations associated with cryptorchidism in rodent testes. Due to significant interspecies differences, our findings should be considered a foundation for understanding responses to cryptorchidism in the mammalian testis rather than a direct model of human cryptorchidism pathophysiology. These findings identify potential phase-specific molecular targets for therapeutic interventions to complement surgical approaches, potentially improving fertility preservation in cryptorchidism patients.

## Data Availability

The original contributions presented in this study are included in the article/Supplementary Materials. Further inquiries can be directed to the corresponding author.

## Supplementary Materials

The following supporting information can be downloaded at: https://www.mdpi.com/article/10.3390/biom15111584/s1, Figure S1: Phase-specific transcriptional signatures in cryptorchidism. Figure S2: Top enriched pathways associated with phase-specific key genes. Figure S3: Pathway intersection analysis of phase-specific key genes. Figure S4: Predicted drug interactions with key genes. Table S1: Testicular development parameters and spermatogenesis data in cryptorchid and control mice at the prepubertal (PND35) and sexually mature (PND70) phases, related to Figure 1 and Figure 2; Table S2: Differentially expressed genes (DEGs) identified in prepubertal (PND35) cryptorchid testes compared with control testes (related to Figure S1); Table S3: Differentially expressed genes (DEGs) identified in sexually mature (PND70) cryptorchid testes compared with control testes (related to Figure S1); Table S4: Gene Ontology (GO) enrichment analysis results of differentially expressed genes in prepubertal (PND35) cryptorchid testes, related to Figure 3A; Table S5: KEGG pathway enrichment analysis results of DEGs in prepubertal (PND35) cryptorchid testes (related to Figure 3B); Table S6: Gene Ontology (GO) enrichment analysis results of differentially expressed genes in sexually mature (PND70) cryptorchid testes, related to Figure 3C; Table S7: KEGG pathway enrichment analysis results of DEGs in sexually mature (PND70) cryptorchid testes (related to Figure 3D); Table S8: Ranked differentially expressed genes (DEGs) from prepubertal (PND35) cryptorchid testes used for GSEA enrichment analysis (related to Figure 3E); Table S9: GSEA enrichment analysis results of prepubertal (PND35) differentially expressed genes via HALLMARK gene sets, related to Figure 3E; Table S10: Ranked differentially expressed genes (DEGs) from sexually mature (PND70) cryptorchid testes used for GSEA enrichment analysis (related to Figure 3F); Table S11: GSEA enrichment analysis results of sexually mature (PND70) differentially expressed genes via HALLMARK gene sets, related to Figure 3F; Table S12: List of Pre-key genes identified through multiple algorithms (MCC, MNC, degree, and closeness) in protein-protein interaction network analysis, related to Figure 7A; Table S13: List of Mat-key genes identified through multiple algorithms (MCC, MNC, degree, and closeness) in protein-protein interaction network analysis, related to Figure 7B. Supplementary Material S1: Quantitative analysis of PI3K-Akt and NF-κB signalling pathway components in cryptorchid testes at prepubertal (PND35) and sexually mature (PND70) phases. Supplementary Material S2: Quantitative analysis of inflammation-related cytokine expression in cryptorchidism at prepubertal (PND35) and sexually mature (PND70) phases. Supplementary Material S3: Quantitative gene expression data by qRT-PCR for key genes at prepubertal (PND35) and sexually mature (PND70) phases. Supplementary Material S4: Complete single-gene GSEA enrichment analysis datasets for prepubertal phase key genes. Supplementary Material S5: Complete single-gene GSEA enrichment analysis datasets for sexually mature phase key genes. Supplementary Material S6: Correlation analysis and functional enrichment data between key genes and germ cell carcinoma-associated genes at prepubertal and sexually mature phases.
