# Supplementary material for: Developmental Phase-Specific Molecular Signatures and Signaling Pathways in Cryptorchidism-Induced Testicular Damage
Source: Biomolecules. 2025 Nov 11;15(11):1584. doi: 10.3390/biom15111584 (PMC12649844; doi:10.3390/biom15111584)
Supplement: Supplementary file 1 [file biomolecules-15-01584-s001.zip › Supplementary material S1--Western blot analysis.zip!/Supplementary material--Western blot analysis/Figure 4. --Western blot analysis/Tagged images and legend explanations of the PI3K-Akt signal transduction pathway.docx]

1.The original WB images for Figures 4A and 4C in the main text of the paper are shown below. Figure 4A is from the left side of the following (1-4) images, PND35; Figure 4C is from the right side of the following (1-4) images, PND70.

(1)

(2)

(3)

(4)
