# Supplementary material for: Developmental Phase-Specific Molecular Signatures and Signaling Pathways in Cryptorchidism-Induced Testicular Damage
Source: Biomolecules. 2025 Nov 11;15(11):1584. doi: 10.3390/biom15111584 (PMC12649844; doi:10.3390/biom15111584)
Supplement: Supplementary file 1 [file biomolecules-15-01584-s001.zip › Supplementary material S1--Western blot analysis.zip!/Supplementary material--Western blot analysis/Figure 5. --Western blot analysis/Figure 5. C/Tagged images and legend explanations of the NF-a╩B signal transduction p.docx]

1. The original WB image of Figure 5C in the main text of the paper is shown below. Figure 5C is derived from the following (1-3) images, PND70.

(1)

(2)

(3)
